# *Notes from the Field:* Mumps Outbreak Associated with Cheerleading Competitions — North Texas, December 2016–February 2017

**DOI:** 10.15585/mmwr.mm6736a6

**Published:** 2018-09-14

**Authors:** Diana Cervantes, Heidi Honza, Daphne Lynch, Nicolette Janoski, Jawaid Asghar, Laura Lockwood, Charles Cohlmia

**Affiliations:** ^1^Texas Department of State Health Services, Public Health Region 2&3, Arlington, Texas; ^2^Collin County Health Services, Mckinney, Texas; ^3^Tarrant County Public Health, Fort Worth, Texas; ^4^Texas Department of State Health Services, Austin, Texas.

On December 6, 2016, Collin County (Texas) Health Care Services (CCHCS) was notified of a suspected mumps case in a woman aged 41 years (patient A), who developed parotitis on December 5. Patient A had attended a cheerleading competition (event 2) 16 days before parotitis onset (Figure). On December 7, CCHCS was notified of a second suspected mumps case in a woman aged 24 years (patient B), with parotitis onset on November 29. Patient B had attended a different cheerleading competition (event 1) 23 days before parotitis onset and worked as a gymnastics instructor at a cheerleading facility (facility A) 2 days before parotitis onset. On December 9, real-time reverse transcription–polymerase chain reaction of buccal swabs performed by the Texas Department of State Health Services (Texas DSHS) confirmed mumps in both patients. After more cases were reported, a call for cases was issued by Texas DSHS. In all, 12 mumps cases (five confirmed and seven probable) in five counties were identified in persons who were nonathlete participants or attendees at three cheerleading competitions or were household contacts of mumps patients.

**FIGURE:**
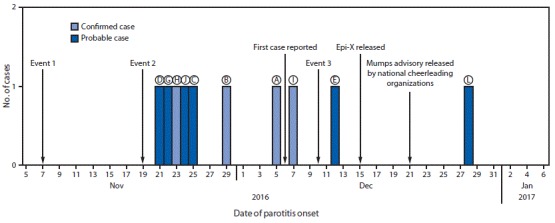
Mumps cases* in persons attending three cheerleading competition events (N = 10), by parotitis onset date^^†^^ — Texas, 2016–2017 **Abbreviation:** Epi-X = CDC’s Epidemic Information Exchange. *Patients indicated by circled letters. Patients F and K are not shown. Both were household contacts of patients and did not attend any cheerleading events before parotitis onset. ^†^ 12–25 days after each event.

Two suspected mumps cases (in patients C and D) were reported to CCHCS on December 9. Patient C, a female aged 15 years and a student of patient B at facility A, reported parotitis onset on November 25 and attendance 19 days earlier at event 1. Patient D, aged 45 years, the parent of another student of patient B’s at facility A, reported parotitis onset on November 21 and attendance at event 1, 15 days before parotitis onset. CCHCS instituted an outbreak investigation focused on contact tracing using CDC guidelines (*1*) and implemented prevention and control measures in collaboration with Texas DSHS, other local health departments, cheerleading facilities, and national cheerleading organizations. Eight additional mumps cases from three other counties were identified (in patients E–L), and all reported attending either event 1 or event 2 during their exposure window (12–25 days before parotitis onset), with the exception of patient F. Patients E and F reported attending another cheerleading competition on December 10, 2016, (event 3) during their infectious period (Figure). However, neither patient F, a household contact of patient G, nor patient K, a household contact of patient H, attended any cheerleading events before parotitis onset.

On December 14, a mumps advisory was issued to staff members and students at facility A. The possibility of multistate exposures at the cheerleading competitions prompted release via CDC’s Epidemic Information Exchange (Epi-X) of a call for cases on December 16. During December 21–22, Texas DSHS partnered with three national cheerleading organizations to release a mumps advisory to the 4,228 registered participants at all three events.

This outbreak resulted in five confirmed and seven probable mumps cases in residents of five counties. Ten cases occurred in females; the median age was 40 years. Among the 10 cheerleading event–associated cases, seven occurred in event staff members and nonathlete, adult attendees. All 12 patients reported having received at least 1 dose of measles-mumps-rubella (MMR) vaccine. Among the eight patients who could provide immunization documentation, five had received 2 MMR doses. CDC performed genotyping on one specimen, identifying mumps virus genotype G.

Although six patients reported attending facility A regularly, it was excluded as an outbreak setting because of inadequate epidemiologic evidence linking cases (i.e., different attendance times and coaches and patients’ parotitis onsets <12 days from one another). Among all 12 patients, six (B, C, D, G, H, and J) attended event 1 and reported symptom onset within the following 12–25 days, implicating event 1 as their likely exposure setting. Three patients (A, E, and I) were likely exposed while attending event 2. One patient (L) attended event 3, but was a household contact of patient E; therefore, the source of exposure could not be established.

Although mumps outbreaks associated with athletic events have been reported (*2*–*4*), this outbreak is the first documented report of mumps transmission during a sporting event with the majority of cases occurring in nonathlete participants or attendees. Receipt of 2 appropriately spaced MMR vaccine doses offers the best protection against mumps; however, transmission can occur at athletic events among athletes, parents, guardians, coaches, and staff members, including appropriately vaccinated persons, underscoring the importance of receiving recommended vaccines to reduce transmission risk or disease severity. Because mumps outbreaks can occur in persons who have received mumps-containing vaccine, contact tracing should include vaccinated persons, and in some outbreak settings, a third dose of MMR vaccine is recommended (*5*).
